# Multifunctional Carbon Fiber Composites: A Structural, Energy Harvesting, Strain-Sensing Material

**DOI:** 10.1021/acsami.2c08375

**Published:** 2022-07-12

**Authors:** Ross Harnden, David Carlstedt, Dan Zenkert, Göran Lindbergh

**Affiliations:** † Department of Engineering Mechanics, KTH Royal Institute of Technology, SE-100 44 Stockholm, Sweden; ‡ Department of Industrial and Materials Science, Chalmers University of Technology, SE-412 96 Gothenburg, Sweden; § Department of Chemical Engineering, KTH Royal Institute of Technology, SE-100 44 Stockholm, Sweden

**Keywords:** carbon fibers, multifunctional composites, sensing, electro-mechanical behavior, piezoelectrochemical transducer effect

## Abstract

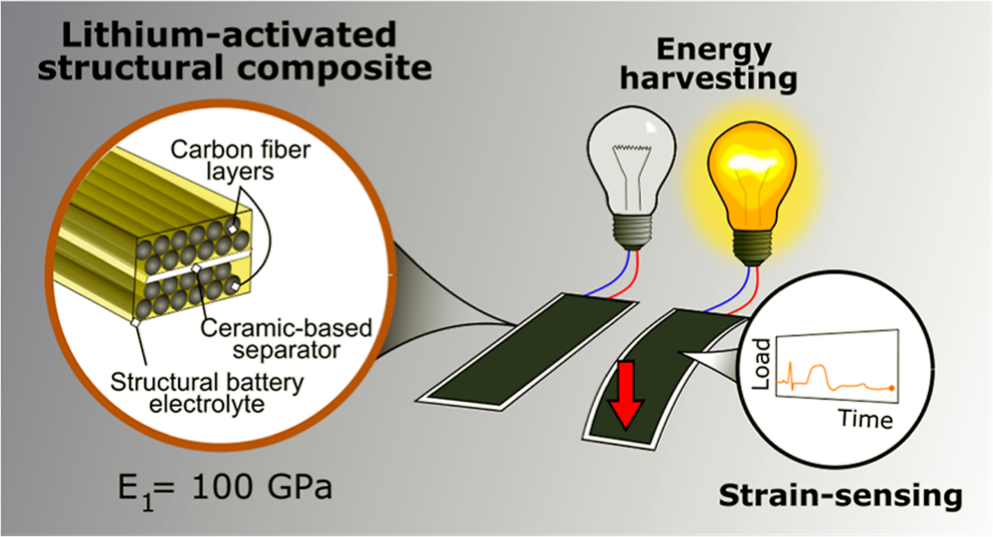

Multifunctional structural materials are capable of reducing system level mass and increasing efficiency in load-carrying structures. Materials that are capable of harvesting energy from the surrounding environment are advantageous for autonomous electrically powered systems. However, most energy harvesting materials are non-structural and add parasitic mass, reducing structural efficiency. Here, we show a structural energy harvesting composite material consisting of two carbon fiber (CF) layers embedded in a structural battery electrolyte (SBE) with a longitudinal modulus of 100 GPa—almost on par with commercial CF pre-pregs. Energy is harvested through mechanical deformations using the piezo-electrochemical transducer (PECT) effect in lithiated CFs. The PECT effect creates a voltage difference between the two CF layers, driving a current when deformed. A specific power output of 18 nW/g is achieved. The PECT effect in the lithiated CFs is observed in tension and compression and can be used for strain sensing, enabling structural health monitoring with low added mass. The same material has previously been shown capable of shape morphing. The two additional functionalities presented here result in a material capable of four functions, further demonstrating the diverse possibilities for CF/SBE composites in multifunctional applications in the future.

## 1 Introduction

Adding functionalities to a material has proven to be an effective way of reducing system level mass in load-carrying structures.^1−3^ Multifunctional materials have the potential to enable lighter components, which are of particular benefit in weight-sensitive applications in the aerospace and automotive sectors, as well as in portable consumer electronics. Ideally, further functionalities should not increase the mass of a structure, nor should they affect its mechanical properties.

Materials that have coupled properties allow further functionalities to be created. For example, piezoelectrics have a mechano-electrical coupling and can be used to convert mechanical motion into electrical energy. This makes it possible to harvest energy from the surrounding environment and even power other material functionalities such as strain sensing.^4^ Piezoelectric materials have previously been integrated into structural composite materials to harvest mechanical energy.^5,6^ However, piezoelectric elements add parasitic mass, show poor mechanical performance, and are most efficient at high frequencies.

For low-frequency motion such as human locomotion, thermal expansions, or tidal flows, energy harvesting using a mechano-electrochemical coupling known as the piezoelectrochemical transducer (PECT) effect seems promising.^7^ The PECT effect is a coupling, resulting in a change in the electrical potential of an electrode when subjected to mechanical strain. PECT energy harvesting in non-structural materials has been carried out using graphite/LiCoO_2_ pouch cells,^8,9^ silicon,^10,11^ aluminum,^12^ black phosphorus,^13^ Prussian blue,^14^ and carbon fibers (CFs),^15^ showing promising results. However, these concepts have all relied on non-structural liquids or gel electrolytes and are therefore not capable of load transfer.

To conceive a structural PECT energy harvester that adds no parasitic mass, the electrode material should be structural and embedded in a matrix capable of transferring load. The matrix must also be ionically conductive to facilitate current flow.

Here, we demonstrate an energy-harvesting structural composite material using a novel combination of materials and applying these to create new functions. The composite consists of two layers of lithiated CFs on either side of a ceramic-based separator embedded in the bicontinuous structural battery electrolyte (SBE).^16,17^ The resulting laminate exhibits high mechanical stiffness but with the added energy-harvesting functionality.

This concept is based on the fact that polyacrylonitrile (PAN)-based CFs are structurally high performing and have proven to be capable of being charged with lithium ions, therefore functioning as an electrode, and is thus in this sense a truly multifunctional material. The matrix used here is denoted a SBE. It is a bicontinuous system composed of one solid and stiff, percolating, polymer phase, and one liquid electrolyte. It can therefore transfer mechanical load between the CFs and is ionically conductive to allow ion transport between electrodes, that is, a multifunctional matrix. The fiber/matrix interface is also multifunctional, providing mechanical adhesion between the fibers and the polymer phase while still allowing ion transport through the interface. This material system has enabled high-performance multifunctional structures to be conceived for energy storage and shape morphing.^2,18^

Previous research has shown that PAN-based CFs in liquid electrolytes exhibit a PECT effect when charged with lithium, sodium, and potassium.^7,19,20^ In PAN-based CFs, lithiation results in the largest PECT response, despite the lower ionic radius.^20^ However, using a liquid electrolyte, it has only been possible to study the PECT response in tension, not in compression. Here, the addition of the SBE allows both compressive and tensile strains to be studied in the CFs. The compressive PECT effect is found to be equal in magnitude but opposite in sign to the tensile PECT effect. This correlates well with an analytical model based on the results of Carlstedt et al.,^21^ which is simplified thanks to the load case and can be expressed as a closed-form solution.

To demonstrate the proof-of-concept for a structural energy harvesting CF composite, a simple bending setup is used. The composite is clamped at one end to form a cantilever, which is deformed to a known constant curvature using a customized clamping jig. In this way, one CF layer is tensioned, while the other is simultaneously compressed. This enables the mechanical strain envelope between the two CF electrodes to be effectively doubled, creating larger voltage and current responses. Using an external lithium metal reference electrode, it is possible to obtain the voltage change in each CF layer independently. The change in open-circuit potential (OCP) created by the PECT effect and short-circuit current (SCC) between the two CF layers are measured during deformation of the cantilever. It is found that both the OCP and SCC increase linearly with the applied mechanical strain. To calculate the available power, a variable external electrical load is connected in series with the composite, and the change in current is measured. The maximum power is obtained when matching the external electrical load with the internal impedance of the composite.

The material demonstrated here is also capable of sensing strain due to the voltage–strain coupling, resulting from the PECT effect. The addition of energy harvesting and strain-sensing functionalities to a structural material, that has previously been shown capable of shape changing,^18^ results in a quadra-functional material. These functionalities combined with excellent structural properties further demonstrate the diverse possibilities for CF/SBE composites in multifunctional applications in the future.

## 2 Materials and Methods

### 2.1 Materials

The CFs used were intermediate modulus T800SC-12K-50C manufactured by Toray Composite Materials America, Inc. Material characterization including X-ray diffraction, high-resolution transmission electron microscopy, and Raman spectroscopy has been performed on this CF previously.^22,23^ The CF tows were spread to a width of approximately 15 mm by Oxeon AB. The SBE consists of a solid phase: bisphenol A ethoxylate dimethacrylate (Sartomer Company, Europe), and 2,2′-azobis(2-methylpropionitrile) (AIBN) and a liquid phase propylene carbonate (PC), ethylene carbonate (EC) (both 99% purity, anhydrous), and lithium trifluoromethanesulfonate (LiTf) (96%) (AIBN, PC, EC, and LiTf supplied by Sigma-Aldrich). A Freudenberg FS 3011-23^24^ separator was placed between the CF layers. Copper foil (17 μm, 99.95% purity) current collectors were attached to the CFs using electrolube silver conductive paint (SCP). During the activation process and for the reference electrode lithium metal foil (0.38 mm, 99.9% purity, Sigma-Aldrich), a Whatman GF/A (260 μm) glass fiber separator paper was used. Nickel foil (25 μm 99.95% purity) was used as a current collector for the lithium foil. For the activation process, pouch cell bags (PET/Al/PE from Skultuna Flexible) were used. Glass fiber end-tabs were manufactured from sheets of cured prepreg (DeltaPreg W105P/DT806). Carbon-film resistors with rated resistances of 10, 100, 1000, and 7000 Ω sourced from NOVA Elektronik Gmbh were used during the energy harvesting experiments.

### 2.2 CF Composite Laminate Manufacturing

**Figure 1:**
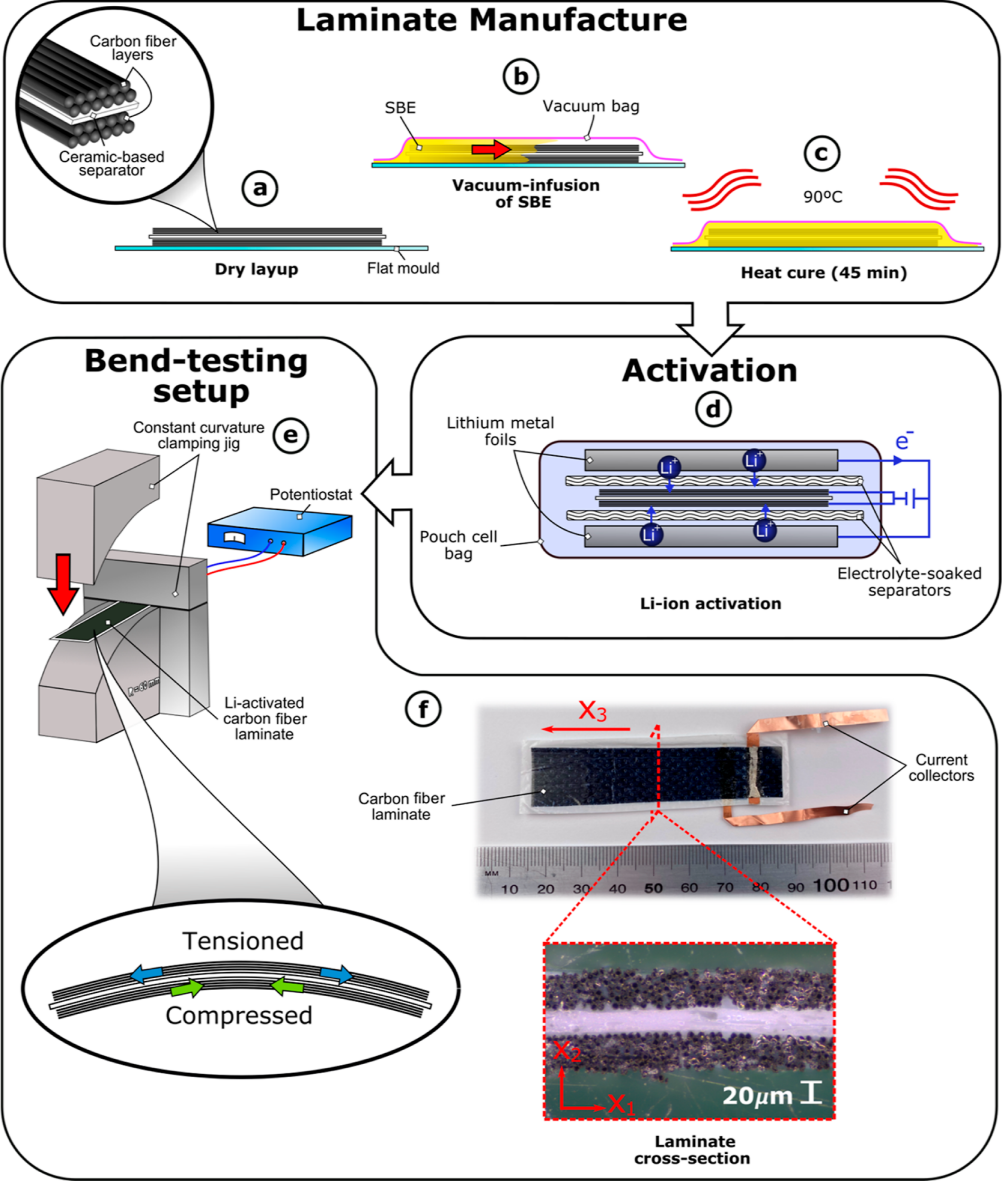
Manufacturing, activation, and testing processes for the composite laminate. (a) Laminate is manufactured using two layers of CF either side of a ceramic-based separator. (b) These layers are vacuum-infused with a SBE. (c) Laminate is heat-cured. (d) Laminate is vacuum-sealed in a pouch cell bag and electrochemically cycled against lithium metal in order to activate it. (e) Laminate is removed from the pouch cell bag and clamped in a cantilever configuration. A clamping jig is then used to deform the cantilever to a known curvature while measuring the voltage and current change between the CF layers using a potentiostat. (f) Macro image and a cross-section of the laminate.

Figure 1a–c illustrates the manufacturing process for the composite laminate. It was manufactured using two layers of CF sandwiched on either side of an electrically insulating separator. The CF samples were prepared using the same method as described previously^20^ where two layers of dry CF were laid on a flat glass mold with a layer of separator between them. Copper current collectors were attached to each layer of CF using SCP. The assembly was dried in a vacuum oven at 60 °C overnight before being sealed in a vacuum bag.

The SBE was mixed inside a glovebox with an inert atmosphere with less than 2 ppm O_2_ and H_2_O at ambient temperature. The mixture consisted of 60.2 wt % BAED, 0.6 wt % AIBN, and 39.2 wt % liquid electrolyte, which is made from 1.0 M LiTf in EC/PC 1:1 wt/wt. The SBE was then vacuum-infused into the dry CF layup and cured in an oven at 90 °C for 45 min.

The cured laminates were removed from the vacuum bag inside the glovebox. The laminates were then placed in a pouch cell between two lithium metal counter electrodes electrically separated by glass fiber separator papers. This was then soaked with around 0.8 mL of the same electrolyte as it is in the SBE (Figure 1d).

### 2.3 Mechanical Testing

Tensile and three-point-bending tests were carried out on the composite laminates after electrochemical testing to establish its longitudinal Young’s modulus. Samples were left under ambient conditions overnight to allow the electrolyte solvents to evaporate. Three-point-bending tests were carried out using an Instron 5567 universal testing machine with a 500 N load cell and a strain-rate of 0.1 mm/min. Samples had a width of 15 mm and a supported length *L* of 12 mm. The measured bending stiffness *D* is given by
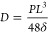
1where *P* is the applied load per unit width and δ is the displacement of the laminate midpoint between the supports. Using laminate theory, it is possible to back calculate the longitudinal elastic modulus of the material *E*_mat_ from the bending stiffness, *D*.^25^ The longitudinal elastic modulus of the CF layers *E*_cf_ is
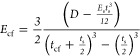
2where *t*_cf_ and *t*_s_ are the thicknesses of the CF layers and separator, respectively, and the longitudinal elastic modulus of the separator is assumed *E*_s_ = 1 GPa. The longitudinal elastic modulus of the material can then be calculated as
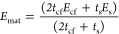
3

For tensile testing, the tabbing material was adhered to the samples (width = 15 mm, and gauge length = 35 mm). An Instron 5567 universal testing machine with a 1000 N load cell and a strain rate of 0.1 mm/min was used. Strain measurements were obtained using a GOM Aramis digital image correlation system. The longitudinal elastic modulus was calculated as the slope of the linear section of the resulting stress–strain curve.

### 2.4 Thickness Measurements

The layer thicknesses of the CF laminates were measured optically. Laminates were dried overnight in a fume hood under ambient conditions to allow the solvents in the electrolyte to evaporate. Sections were then cut manually using a scalpel, and the resulting sections were potted in an epoxy-based potting compound. The surfaces were polished and then photographed using an Olympus BX53M light microscope with Olympus Stream Basic (v2.3.3) software.

### 2.5 Activation Prior to Experimentation

To activate the CF layers with lithium ions, the laminate was placed between two sheets of lithium metal foil with glass fiber separators preventing electrical contact (see Figure 1d). These layers were then vacuum sealed in a pouch cell with the liquid electrolyte. The samples were activated by charging and discharging the CFs against the lithium metal between 0.002 and 1.5 V versus Li/Li^+^ at a current density of 28 mA/g, based on the mass of CFs. The charging/discharging was performed using a Biologic VSP potentiostat. The maximum capacity achieved was 160 mA h/g at a C-rate of around 0.1 C. On the 12th cycle, the CFs were charged to approximately 60% degree of lithiation, approximately 105 mA h/g (see Figure S1), since the largest PECT response has previously been shown to occur at approximately this state of lithiation.^15^

### 2.6 Composite Voltage–Strain Coupling and Energy Harvesting

Prior to experimentation, the CF layers were connected to each other via an external circuit for at least 2 h, to allow any residual lithium concentration to dissipate.

All voltage–strain coupling and energy harvesting measurements were carried out in the glovebox with less than 2 ppm O_2_ and H_2_O at ambient temperature. To create bending with a lengthwise constant curvature, a custom-made two-part 3D-printed clamping jig was used (see Figures 1e and S2). For the independent voltage measurements of the two CF electrodes, a strip of lithium metal was inserted into a notch in the lower section of the clamping jig and separated from the cantilever using an electrolyte-soaked glass fiber paper separator.

For the voltage–strain coupling experiments, the cell was left to rest for 5 min to allow a reference voltage for each electrode to be recorded. After this, the top section of the jig was clamped down by hand on top of the composite cantilever, causing the cantilever to bend into a constant curvature. During these measurements, no current was applied.

For the energy harvesting experiments, different external electrical loads were connected. Carbon film resistors were soldered on to a stripboard, along with appropriate connectors. These were connected in series with the potentiostat and the CF laminate. The values of the resistances including the cabling were measured using a multimeter, and the true resistance values were used to calculate the voltages using Ohm’s law.

A GoPro Hero 5 camera was used to film all voltage strain coupling and energy harvesting experiments.

All voltage and current signals were filtered using a third-order Daubechies wavelet filter to minimize noise (see Figure S3 for examples). Average peak and trough values were calculated as well as standard deviations. The change in voltage/current is then given by the difference between the average peak and trough values.

## 3 Modeling of the PECT Effect

The voltage–strain coupling resulting from the PECT effect was modeled analytically based on the assumed coupling between the chemical potential of lithium in the CFs and the mechanical stress state, using the Larché-Cahn potential.^26,27^ The theory accounts for the transversely isotropic CFs^21^ where isotropy pertains to the cross-section defined by Cartesian coordinates *x*_1_ and *x*_2_, and *x*_3_ is along the fiber (see Figure 1f).

The change in equilibrium potential of the CF versus lithium metal (Δ*V*_0_) is a function of the change in the strain state (Δ**ϵ**), giving the generalized form for the voltage–strain coupling
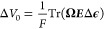
4where ***E*** is the transversely isotropic elasticity matrix, **Ω** represents the normalized (transversely isotropic) change in volume of the CF as a function of the lithium concentration, and *F* is Faraday’s constant. Note that here the equilibrium potential is not only related to the mean stress as in the case of isotropy. It is assumed that the lithium concentration in the CFs remains constant and that the applied strains are small and within the linear-elastic regime of the composite.

For changes in uniaxial strain (Δϵ_33_), a simplified analytical expression can be obtained. The applied strain is mainly carried by the CFs, and it is assumed that the radial fiber deformation is unconstrained (motivated by the difference in stiffness of CF vs SBE). Under these conditions, eq 4 simplifies to
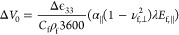
5where λ = 1/[(1 + ν_f,⊥_) (1 – ν_f,⊥_ – 2*Y*ν_f,∥_^2^)] and *Y* = *E*_f,⊥_/*E*_f,∥_. The elastic moduli and Poisson’s ratio of the fiber parallel and perpendicular to the fiber direction are denoted *E*_f,∥_, *E*_f,⊥_ and ν_f,∥_, ν_f,⊥_, respectively. Furthermore, *C*_f_ is the specific capacity of the CF, ρ_f_ is the fiber density, and α_∥_ represents the reversible longitudinal expansion coefficient of the CF for the assumed specific capacity.^28^ Note that the strain in the fiber direction (ϵ_33_) in each CF layer is represented by the average strain caused by the applied bending moment. The complete derivation of the voltage–strain coupling and the utilized material parameters are available in the Supporting Information.

## 4 Results

### 4.1 Mechanical and Physical Properties of the Laminate

**Figure 2:**
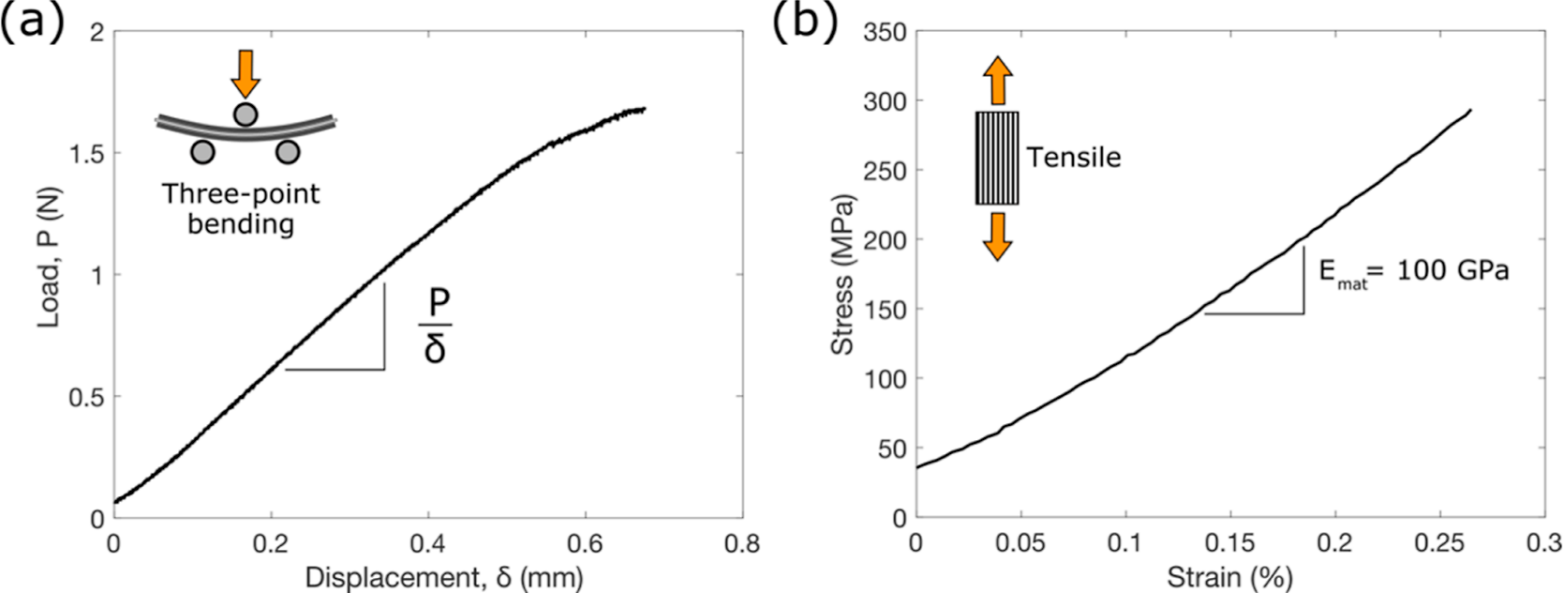
Mechanical testing of the laminate. (a) Example force–displacement curve from three-point bending of the CF composite laminate and (b) example stress–strain curve from tensile testing of the CF composite laminate.

The resulting laminate has a longitudinal (fiber direction) elastic modulus of 100 GPa obtained from both bending and tensile tests of the laminate. An example of a load–displacement profile from bending tests and a stress–strain profile from tensile testing can be seen in Figure 2. Experimental data from the tests are given in Table S1.

The density of the composite laminate was derived using the volume fractions and densities of the constituent components. The density of the CFs is given as 1.8 g/cm^3^,^29^ the density of the separator is given as 1.435 g/cm^3^,^24^ and the SBE has a density of 1.23 g/cm^3^. The average volume fraction of CF to SBE in the CF layers is 49%, while the volume fraction of the separator to SBE in the separator layer is 45%.^24^ This gives an average density of the laminate of 1460 kg/m^3^. For comparison, a typical CF unidirectional prepreg has a modulus in the range 130–180 GPa and density around 1600 kg/m^3^, while aluminum has a modulus of 69 GPa and density of 2700 kg/m^3^. After potting and polishing the samples, layer thicknesses of 32 μm for the CFs and 20 μm for the separator were obtained using microscopy (see Figure 1f, S4 and S5, and Table S2). It should be noted that variations in thickness contribute to variations in the calculated mechanical properties of the different samples.

### 4.2 PECT Effect in Tension and Compression

**Figure 3:**
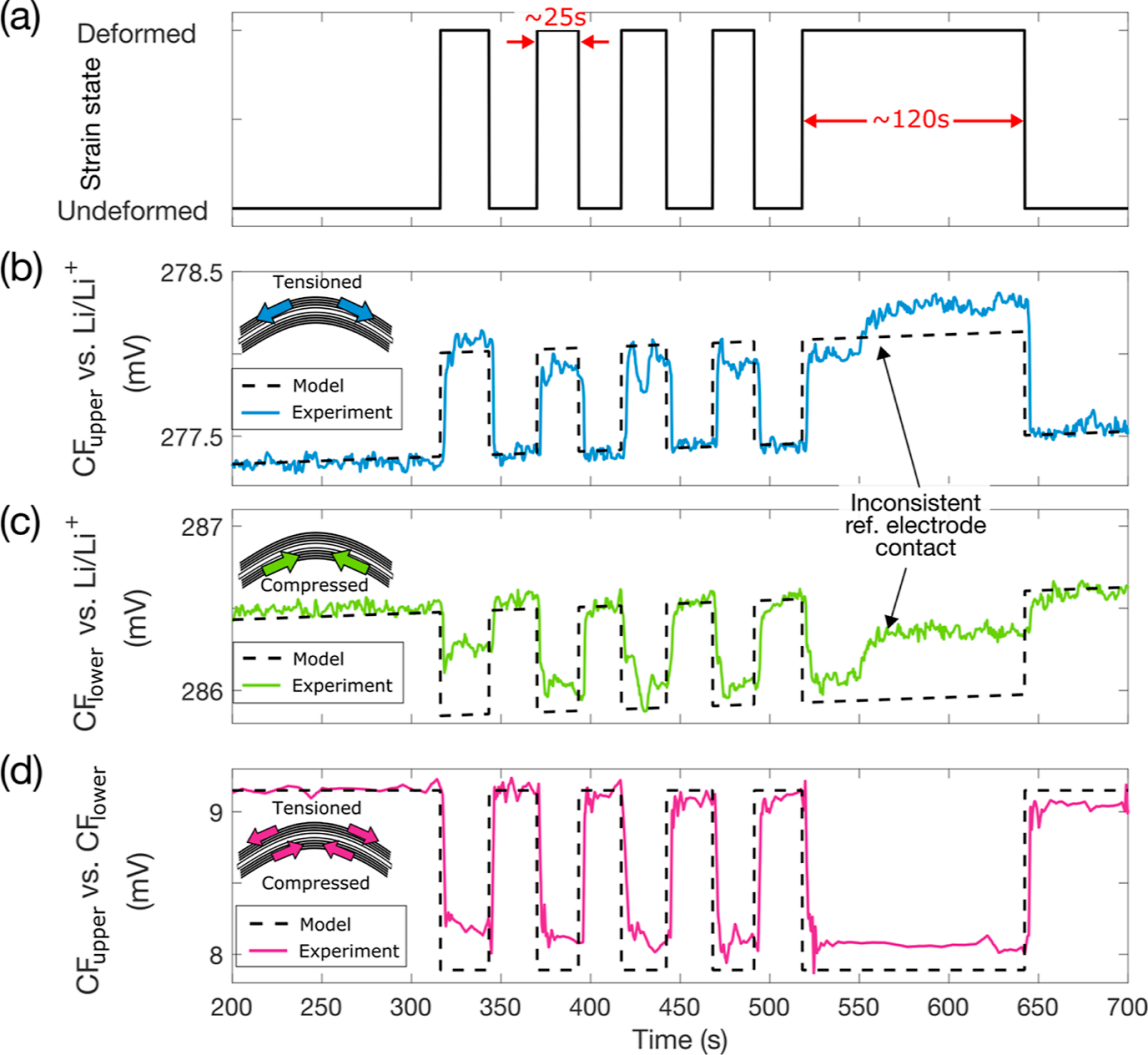
PECT measurements. (a) State of strain during cyclic bending of the cantilever. (b,c) Voltage of the cantilever’s upper and lower CF layer, respectively, vs a lithium metal reference electrode. A drift in the reference electrode of approximately 0.2 mV was observed during the measurement. This was accounted for in the modeling using a linearly increasing initial voltage with a gradient of approximately 4 × 10^–4^ mV/s. (d) Voltage difference between the cantilever’s two CF layers. Dashed lines are theoretical predictions.

After activation, the laminate was removed from the pouch cell bag and wrapped in a thin layer of low-density polypropylene (thickness ≈ 15 μm) to prevent the liquid phase of the SBE evaporating. The laminate was clamped at one end to form a cantilever, and the two current collectors were connected to a potentiostat to apply and measure current and voltage. The two-part 3D-printed clamping jigs of known constant curvature were used to mechanically deform the cantilever along the fiber direction (see Figure 1e). Deformations were held in place with the clamping jig for approximately 25 s before being released for 25 s. This was repeated four times, before a final deformation was held for approximately 120 s. The strain state is shown in Figure 3a and is compressive in one layer and tensile in the other with equal amplitudes. In the deformed state, the strain in the fiber direction varies linearly through the laminate thickness. Here, the average strain change in each CF layer (Δϵ_33_) is used to represent the deformed strain state (see Figure S6). Figure 3b shows the upper CF electrode being repeatedly deformed to a radius of 30 mm, corresponding to an average tensile strain change of Δϵ_33_ = 0.09%. On deformation, a clear rise in the potential from the steady-state is observed, with a magnitude of approximately 0.52 ± 0.14 mV versus the Li metal counter electrode. Figure 3c shows the lower CF layer during the same deformation but with Δϵ_33_ = −0.09%. There is a clear drop in the potential from the steady state, with a magnitude of approximately 0.49 ± 0.13 mV versus Li. The change in voltage occurs as fast as the deformation is applied, as can be seen from Movie S1. The compressive PECT effect is thus equal in magnitude but opposite in sign to the tensile PECT effect.

Small variations in the potential can be seen in Figure 3b,c, particularly during the longer deformation. This is thought to be caused by the inconsistency of contact with the lithium metal reference electrode.

Figure 3d shows the same testing while measuring the electrical potential between the two CF layers. The voltage change is around 1 mV. This equals the sum of the magnitudes of the two independently measured electrode potentials. Here, in the absence of a reference electrode, the voltages appear to remain more consistent upon deformation, and there is no tendency for the voltages to return to the steady state. Applying bending deformations thus effectively doubles the obtainable voltage change. This effect enables the measurement of bending strains within the material, with no parasitic components.

For a strain of 0.09%, the predicted voltage change versus Li/Li^+^ is 0.63 mV, as shown by the dashed lines in Figures 3b,c, which corresponds well to experimental observations. This leads to a voltage difference between the two CF layers of 1.26 mV when strained to ±0.09%, which also agrees well with experimental data seen in Figure 3d. The predicted changes are slightly higher than the experimental observations, a discrepancy which is thought to be caused by model simplifications, for example, the imposed boundary conditions and utilized material data (see Table S3).

The PECT effect in direct tension was tested to verify the assumption of using average strains to represent bending deformations. This was measured using a previously described methodology.^7,19,20,28^ A cyclic tensile strain of 0.11% was applied to a lithiated CF bundle using a tensile tester, and the PECT response was measured (Figure S7). The magnitude of the potential change is 0.52 mV (see Figure S8), which is consistent with previous work.^7,19,20,28^ This shows that the average strains being applied using the clamping jigs can be compared to those applied using direct tensile strains.

### 4.3 Short-Circuit Current Measurements

By preventing current flow when the laminate is deformed, the PECT effect creates a change in OCP. Conversely, by connecting the two CF electrodes via an external circuit and enforcing a 0 V potential, a current will flow when the laminate is deformed, known as the SCC. The OCP and SCC give the upper limits of available voltage and current change, respectively, upon strain application. The coupled nature of the electrochemical and mechanical systems enables energy harvesting.

The OCP and SCC between the two CF layers were measured for various applied average strain differences, given by 2|Δϵ_33_|. For example, with one layer tensioned to 0.09% strain, and the other compressed to −0.09% strain, the strain difference becomes 0.18%. A range of strains was applied using four different clamping jigs giving strain differences equal to 0.04, 0.06, 0.08, and 0.18%. Both upward and downward bending were applied by inverting the jigs. For the OCP measurements, the two CF electrodes were connected to a potentiostat, and no current was allowed to flow while the voltage was measured. For the SCC, the potentiostat was used to hold the voltage between the CF layers at 0 V, and the current was measured. The cantilever was deformed first in downward bending and then in upward bending, with strains applied for approximately 10 s, with 10 s intervals between.

**Figure 4:**
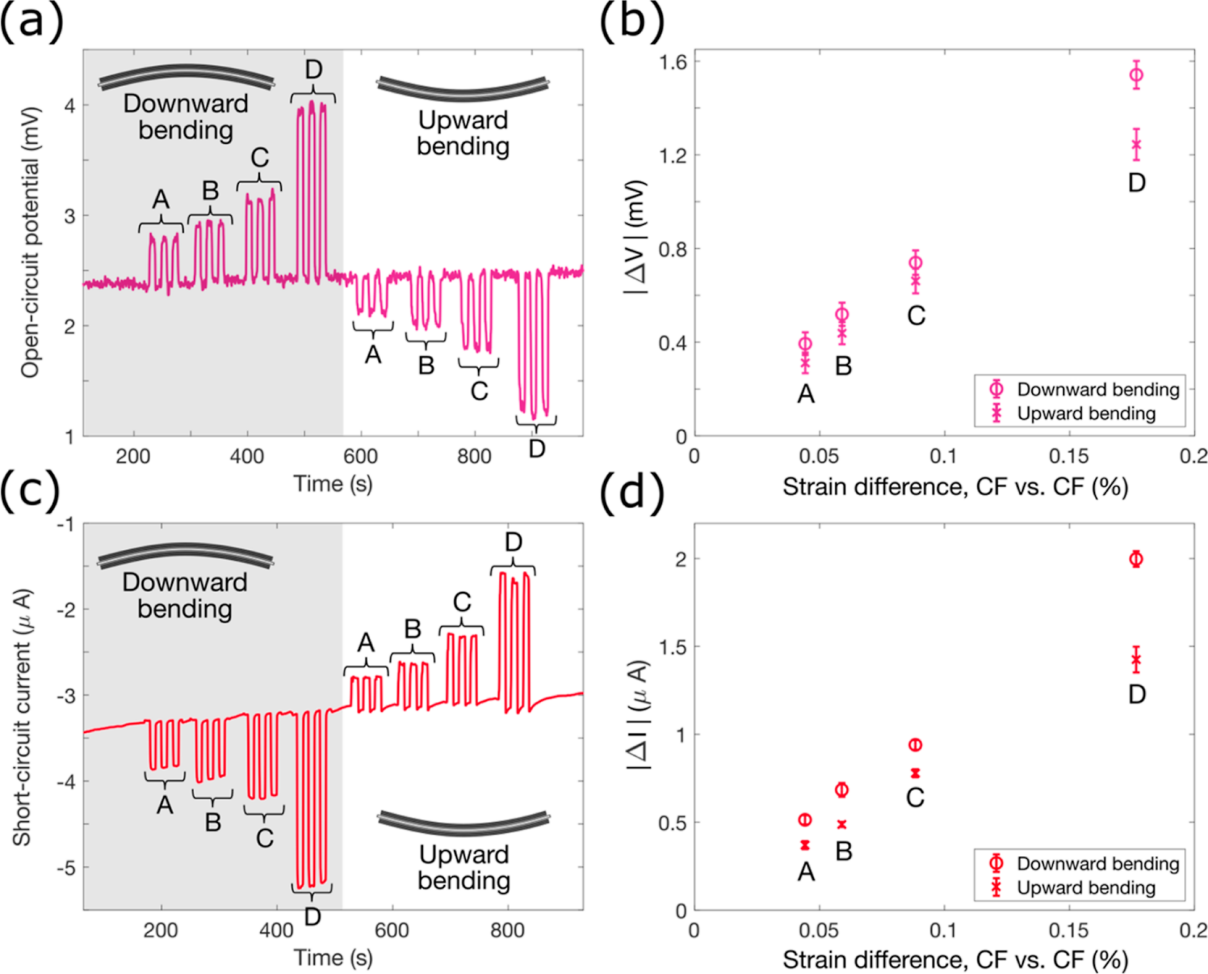
OCP and SCC response at various bending curvatures. (a) OCP between the two CF layers for varying strain differences in both upward and downward bending. (b) Average magnitude of OCP response between the two CF layers for varying strain differences. (c) SCC between the two CF layers for varying strain differences in both upward and downward bending. (d) Average magnitude of SCC response between the two CF layers for varying strain differences. The CF laminate cantilever used here had an active length of 42 mm and a width of 15 mm, giving a total mass of 78.5 mg. The active CF electrode mass is 43.6 mg.

Figure 4a and Movie S2 show the OCP responses across the range of applied strains for both upward and downward bending. The OCP increases with increased applied strain and is opposite in sign when changing from downward to upward bending. Figure 4b shows the magnitude of the average OCP change for each applied strain. Error bars represent the summation of the standard deviations of the OCP in the unstrained and strained states. The OCP response is linear with strain, reaching a maximum of approximately 1.5 mV for a strain difference of 0.18%. When reversing the strain direction from upward to downward bending, the sign of the OCP response changes from positive to negative, although the magnitude of response is largely the same. The OCP response is thus a direct measurement of the average level of strain in the material, as well as the bending state.

Figure 4c and Movie S3 show the SCC response upon application of strains, whereas Figure 4d shows the magnitudes of the average SCC change, with error bars representing the summation of the standard deviations of the SCC in the unstrained and strained states. The magnitude of the SCC response is linear with strain and reaches a maximum of approximately 2 μA for a strain difference of 0.18%. The SCC follows the same pattern as the OCP, with the response changing sign when going from upward to downward bending. The magnitudes of the current changes are slightly lower for upward bending. This could be due to the small residual negative current between the CF layers. This creates ion distribution gradients in the electrochemical system, which would slightly amplify the more negative response and slightly dampen the more positive response.

### 4.4 Energy Harvesting

Measurements of the OCP and SCC give the theoretical upper limit of available power, with the product of the two giving the idealized power available. This is the power often reported for similar energy harvesters.^10,12,13^ In this case, the maximum idealized power available is about 69 nW/g based on the active CF electrode mass. However, since there is no potential difference when the SCC is measured, and no current flow when the OCP is measured, no power is generated. In order to measure the actual power available, it is necessary to have a known electrical load in the circuit.

**Figure 5:**
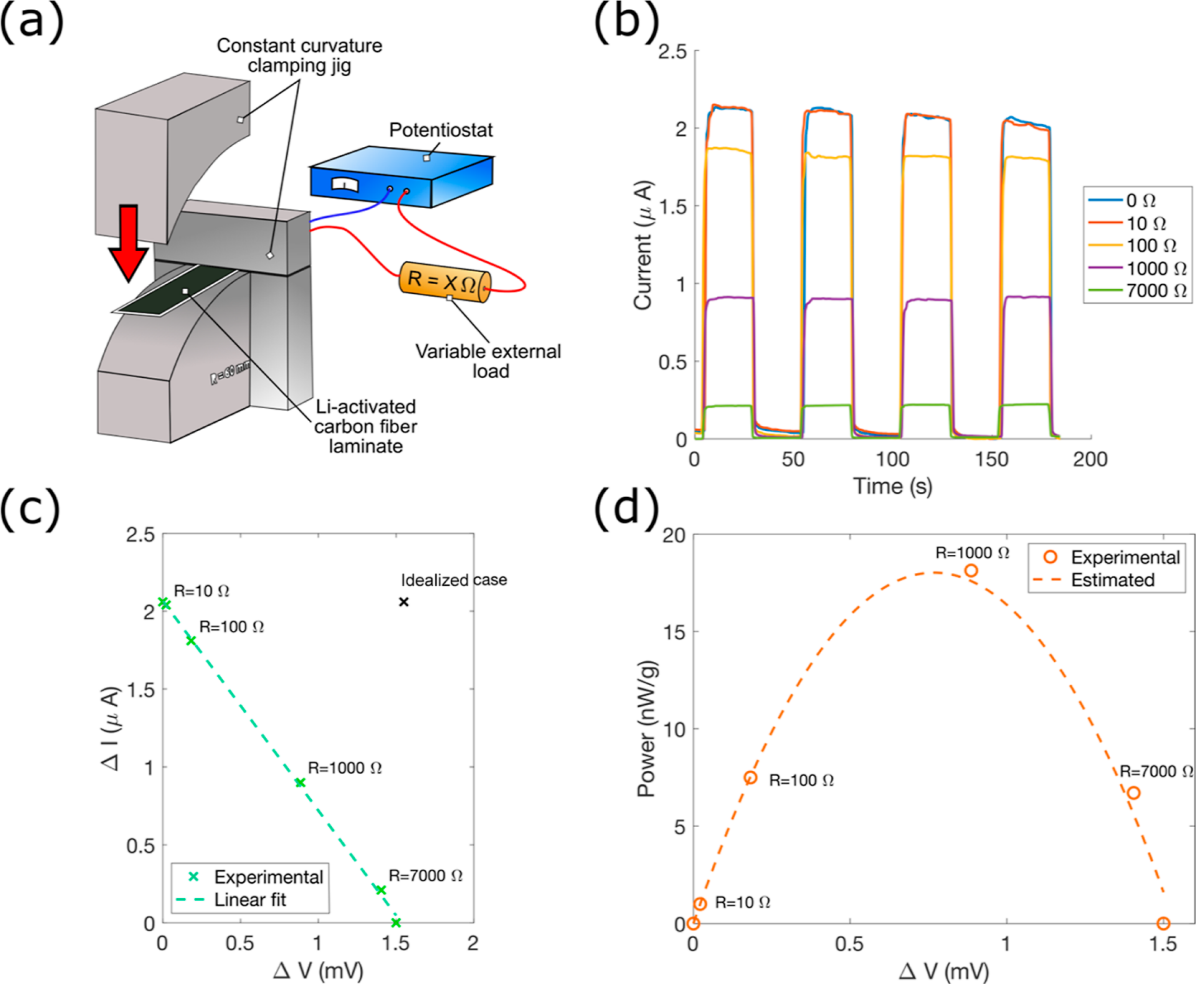
Energy harvesting results. (a) Experimental setup to measure the power available for energy harvesting. Different external electrical loads were connected in series with the CF laminate and potentiostat while the change in current was measured. (b) SCC between the two CF layers during bending, with a strain difference of 0.18%. (c) Current–voltage profile during bending with various external electrical loads and a linear fit of the measured responses. (d) Gravimetric power profile for energy harvesting with various external electrical loads and an active CF electrode mass of 43.6 mg.

The methodology reported by Preimesberger et al.^30^ is used whereby various resistors were connected in series in the electrical circuit, as shown in Figure 5a. Resistors of 10, 100, 1000, and 7000 Ω were used. The highest available applied strain was used, corresponding to a strain difference of 0.18%. A potentiostat was used to enforce 0 V between the CF layers and measure the change in current upon deflection of the composite cantilever. The corresponding voltage change across the resistor was calculated using Ohm’s law. The product of the voltage change and the current is the output power.

Figure 5b shows the current profile upon strain application, with increased external resistance resulting in a lower current response. Figure 5c shows the resulting current–voltage curve. As expected, the relationship between current and voltage is linear for small perturbations.^31^ A linear curve fit is shown in Figure 5c. The power can then be calculated and is shown in Figure 5d. The theoretical power can be calculated by integrating the linear curve fit from Figure 5c with respect to the voltage. The maximum measured power is around 18 nW/g based on active CF electrode mass. The fill factor, which is defined as the ratio between the idealized power and the maximum available power, is therefore around 26%, which is similar to that reported elsewhere.^30^ The maximum power is obtained using a 1000 Ω external resistor, which approximately matches the internal resistance between the CF layers of 860 Ω which was found using electrical impedance spectroscopy (see Figure S9). This agrees with the maximum power transfer theorem,^32^ as well as previous findings.^10^

## 5 Discussion

This research has demonstrated two additional functionalities in a CF composite laminate: energy harvesting and strain sensing. The power measured experimentally compares favorably with the other published literature on PECT energy harvesters such as lithiated aluminum and sodiated black phosphorus.^12,13^ However, these studies use non-structural materials using liquid electrolytes and require significantly higher applied strains.

The CF laminate demonstrated here incorporates an SBE, producing a structural material with a specific stiffness considerably higher than aluminum and in line with a commercial CF pre-preg. Strain sensing is possible due to the voltage–strain coupling created by the PECT effect, with a linear relationship between OCP and mechanical strain. The theoretical framework for estimating the voltage–strain coupling was in good agreement with the experimental observations. Due to the noise in the voltage signal (about 0.1 mV for a filtered signal, FigureS 3), a lower bound for strain measurements would be around 0.01%.

The longevity of the voltage and current responses have not been examined here. However, it is thought that the CFs themselves should not suffer significant structural damage during repeated bending as the strains used here are very low. The effect of lithium insertion on the mechanical properties of the same CFs has been tested previously, with minimal mechanical degradation observed even after 1000 cycles.^33^ The mechanical properties after electrochemical cycling of a single layer of CFs embedded in the SBE have been tested previously, showing no degradation in stiffness or strength.^34^ Considering that strains applied in the energy harvesting experiments are very low (0.09%), this would be very unlikely to affect the mechanical properties over time. Other PECT energy harvesters exhibit relatively good long-term stability over repeated cycling,^10,12,13^ suggesting that the response should be stable over repeated mechanical strain cycles. The calendar life of the electrochemical system is most likely the limiting factor in our case, given the small amount of charge transfer. This would be dictated by how well the system could be isolated from the external environment (e.g., from moisture ingression).

Since this is a very first attempt to create a stiff energy harvesting material, the material constituents or the material assembly are far from being optimized. A number of things could be done to improve the magnitude of the current and voltage responses, allowing more energy to be harvested. By doubling the strain, it should be possible to double the change in OCP and SCC. This would result in four times the power since within the strain envelope used here, the power scales with the square of the strain. The strains could probably be increased even further provided mechanical failure does not occur. Differences in the PECT response, although rather small, have been measured previously for two different CFs.^15^ It is thus possible that there are other CFs with higher PECT responses, which could improve both the sensing capability and the harvested power. Smaller diameter CFs would lead to faster diffusion, giving higher rate capability and reducing overpotentials. The SBE used has not yet been optimized and further development work could lead to an order of magnitude improved ionic conductivity. By adjusting the porous polymer structure of the SBE, the tortuosity could be reduced, improving ionic conductivity. By using different solvent/salt combinations, it would most likely be possible to improve the transport properties of the liquid electrolyte and hence increase the current response of the energy harvester. This would also extend the frequency envelope of the harvester at higher frequencies, although PECT-based energy harvesting is still limited to low frequencies due to the ion diffusion process, as discussed elsewhere.^10^

The experimentation conducted in this article was carried out in a dry argon atmosphere. By properly encapsulating the CF laminate using films with appropriate barrier properties, such as ultrathin glass,^35^ the laminate could operate under ambient conditions.

The ability to harvest energy from the surrounding environment is an important complementary ability to energy storage.^36^ A low-to-moderate frequency structural energy harvester is particularly useful in autonomous applications where weight is sensitive, such as unmanned aerial vehicles, satellites, and medical applications. To exploit the multifunctionality, devices that incorporate a structural function along with a strain sensing function would be ideal. Sensing strain in real time minimizes the oversizing of structures and improves the safety and maintenance routines.^37^ Lithiated CFs could be used to replace other structural health monitoring systems that add parasitic mass and adversely affect mechanical properties,^38^ such as optical fibers, piezoelectrics, and piezoresistive materials.^39−45^ The same structural material can be used for shape-morphing,^18^ and with the addition of a positive electrode layer could also function as a structural battery.^2^

The analytical model of the voltage–strain coupling has the potential to aid design of PECT energy harvesters and strain sensors in future, as well as of anisotropic battery electrodes under mechanical strain.

## 6 Conclusions

The structural CF composite laminate presented here has a longitudinal modulus almost on par with commercial CF pre-preg materials that have previously been shown to be capable of shape changing.^18^ The two further functionalities demonstrated here result in a material that performs four functions simultaneously: load bearing, shape changing, energy harvesting, and strain sensing. The research demonstrates the diverse possibilities for CF/SBE composites in multifunctional applications in the future.

## Supporting Information Available

Additional experimental details, materials, and methods, including photographs of materials (PDF)
Tensile and compressive OCP responses (MP4)
OCP between the two CF layers for various deformations (MP4)
SCC between the two CF layers for various deformations (MP4)
