# Supplementary material for: Multifunctional Carbon Fiber Composites: A Structural, Energy Harvesting, Strain-Sensing Material
Source: ACS Appl Mater Interfaces. 2022 Jul 12;14(29):33871–80. doi: 10.1021/acsami.2c08375 (PMC9335530; doi:10.1021/acsami.2c08375)
Supplement: Supplementary file 1 — am2c08375_si_001.pdf [file am2c08375_si_001.pdf]

**Supporting Information**

# Multifunctional Carbon Fiber Composite: A Structural, Energy Harvesting, Strain-Sensing Material

*Ross Harnden [a], David Carlstedt [b], Dan Zenkert [a]*, Göran Lindbergh [c]*

[a] Department of Engineering Mechanics, KTH Royal Institute of Technology, SE-100 44 Stockholm, Sweden

[b] Department of Industrial and Materials Science, Chalmers University of Technology, SE-412 96 Gothenburg, Sweden

[c] Department of Chemical Engineering, KTH Royal Institute of Technology, SE-100 44 Stockholm, Sweden

* Corresponding author: danz@kth.se

# Contents

*Activation cycling curves*

**Figure S1.** Lithiation and delithiation of the composite laminate. Electrochemical cycling was conducted against lithium metal foil, between 1.5 V and 0.002 V vs $Li/Li^+$, at a current density of 28 mA/g. A final capacity of 105 mAh/g was used for the subsequent bend testing. The active mass of the CFs was 70.1 mg.

*Clamping jigs used in the experiments*

**Figure S2.** Renderings of clamping jigs used during experimentation.

*Filtering of voltage profiles*

**Figure S3.** Examples of voltage profiles before and after filtering.

*Mechanical testing of CF laminates*

**Table S1**. Experimentally measured longitudinal elastic moduli and bending stiffnesses for 3 laminates.

*Thickness measurements*

**Figure S4**. Example cross-section of laminate with measurements

**Figure S5.** Example cross-section of laminate with measurements.

**Table S2.** Mean thicknesses from 3 different CF laminate samples, along with standard deviations.

*Strain within CF laminate*

**Figure S6.** Strain variation through CF laminate thickness in undeformed and deformed states.

*Derivation of voltage-strain coupling*

**Table S3**. Material parameter values.

*Direct tensile measurement of voltage-strain coupling*

**Figure S7.** Measurement of the voltage-strain coupling using a direct tensile strain of 0.11% applied by a microtester on a CF tow, in liquid electrolyte, in a pouch cell bag against lithium metal.

*Direct tensile measurement of voltage-strain coupling*

**Figure S8.** Comparison of voltage-strain coupling measurement using bend testing, and direct tensile measurements.

*Electrical Impedance Spectroscopy*

**Figure S9.** Nyquist plot following EIS measurements carried out between CF layers. The intercept at low frequency can be used to estimate the internal resistance of the cell, and in this case corresponds to approximately 860 Ω.

**Movie S1.** Video showing the cantilever being deformed in the downward direction to a radius of curvature of 30 mm, corresponding to an average strain magnitude of 0.09%. The OCP measured between the upper and lower CF layers versus a lithium metal reference electrode is shown. This allows the tensile and compressive OCP responses to be observed respectively. The OCP between the two CF layers is also shown, corresponding to an average strain difference of 0.18%. The first four deformations are held for approximately 25 seconds, with 25 seconds in between each deformation. The last deformation is held for approximately 120 seconds. The video is shown in real time for the first deformation, and then sped up by 10 times for subsequent deformations.

**Movie S2.** Video showing the OCP between the two CF layers for various deformations. A potentiostat is used to measure the voltage difference between the CF layers. Clamping jigs that result in an average strain difference between the CF layers of 0.04%, 0.06%, 0.08% and 0.18% are used. Three repetitions of each strain are carried out, with deformations held for approximately 10 seconds, before being released for 10 seconds. Downward bending of the cantilever is shown first, followed by upward bending. The video is shown in real time for the first three deformations, and is then sped up by 10 times for subsequent deformations.

**Movie S3.** Video showing the SCC between the two CF layers for various deformations. A potentiostat is used to hold the voltage difference between the CF layers at 0 V, and measure current. Clamping jigs that result in an average strain difference between the CF layers of 0.04%, 0.06%, 0.08% and 0.18% are used. Three repetitions of each strain are carried out, with deformations held for approximately 10 seconds, before being released for 10 seconds. Downward bending of the cantilever is shown first, followed by upward bending. The video is shown in real time for the first three deformations, and is then sped up by 10 times for subsequent deformations.

*References used in the supporting information*

**Activation cycling curves**

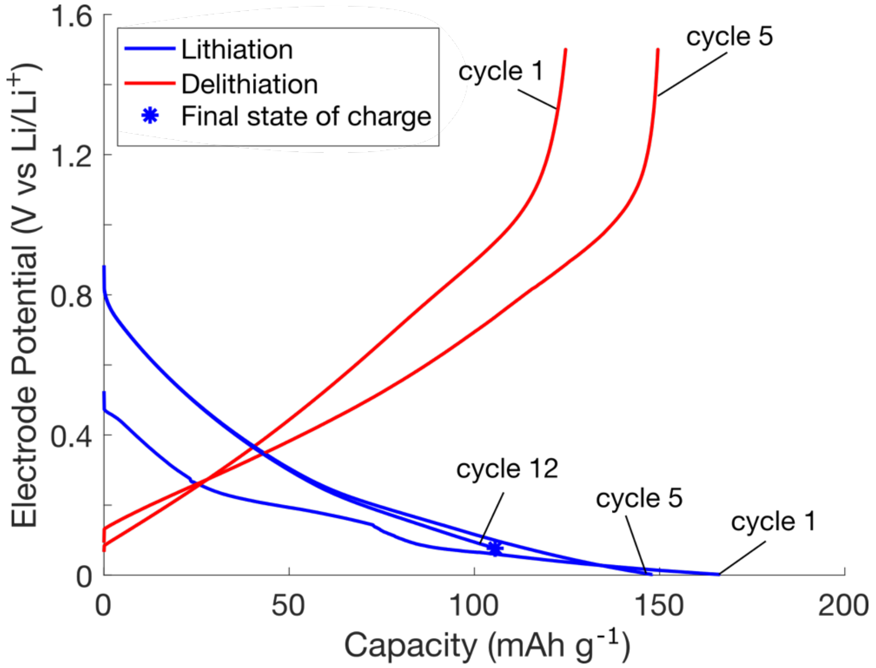


**Figure S1**. Lithiation and delithiation of the composite laminate. Electrochemical cycling was conducted against lithium metal foil, between 1.5 V and 0.002 V vs $Li/Li^{+}$, at a current density of 28 mA/g. A final capacity of 105 mAh/g was used for the subsequent bend testing. The active mass of the CFs was 70.1 mg.

**Clamping jigs used in the experiments**

The clamping jigs were 3D printed using polyactic acid (PLA) and an Ultimaker 3 printer. Several sets of jigs were used, with varying radii of curvature, enabling different levels of strain to be applied. One set of jigs was printed with a small cut-out in the lower clamp that allowed a Li-metal reference electrode to be placed directly under the CF composite cantilever (see **Figure S2**).

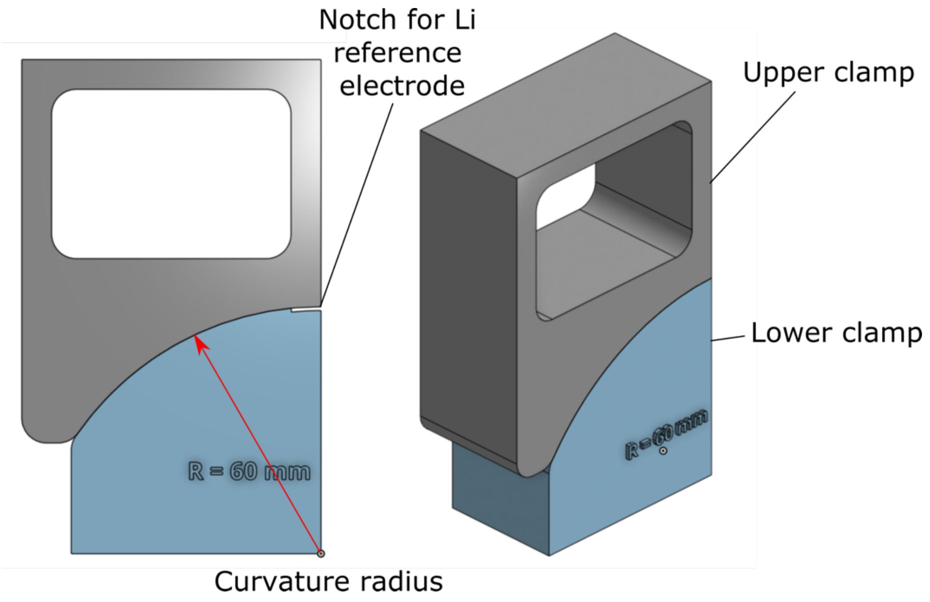


**Figure S2.** Renderings of clamping jigs used during experimentation.

**Filtering of voltage profiles**

All voltage signals were filtered using a third-order Daubechies wavelet filter during post-processing to minimize noise. This filtering was chosen as it did not change the desired characteristics of the waveforms, and is computationally efficient.

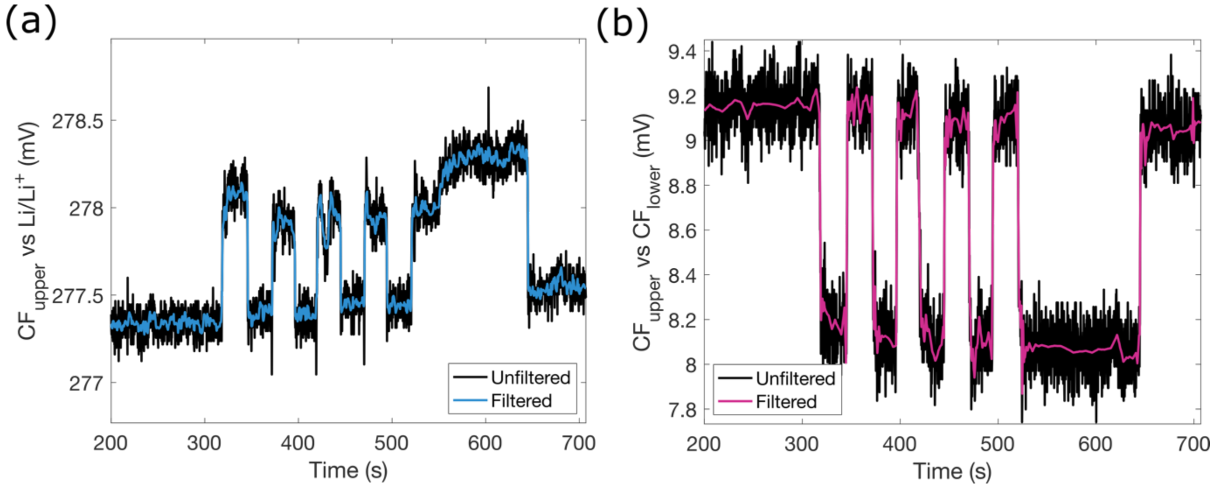


**Figure S3.** Examples of voltage profiles before and after filtering.

**Mechanical testing of CF laminates**

**Table S1**. Experimentally measured longitudinal elastic moduli and bending stiffnesses for 3 laminates.

| | **Material longitudinal elastic modulus, $E_{mat}$ (GPa)** | **Bending Stiffness, D (N/m)** | **Material longitudinal elastic modulus from bending, $E_{mat}$ (GPa)** |
|---|---|---|---|
| Sample 1 | 100,66 | 0,0067 | 118,83 |
| Sample 2 | 102,24 | 0,0070 | 103,38 |
| Sample 3 | 100,91 | 0,0065 | 95,05 |
| **Mean** | **101,27** | **0,0067** | **101,54** |
| Standard deviation | 0,85 | 0,0002 | 18,28 |

**Thickness measurements**

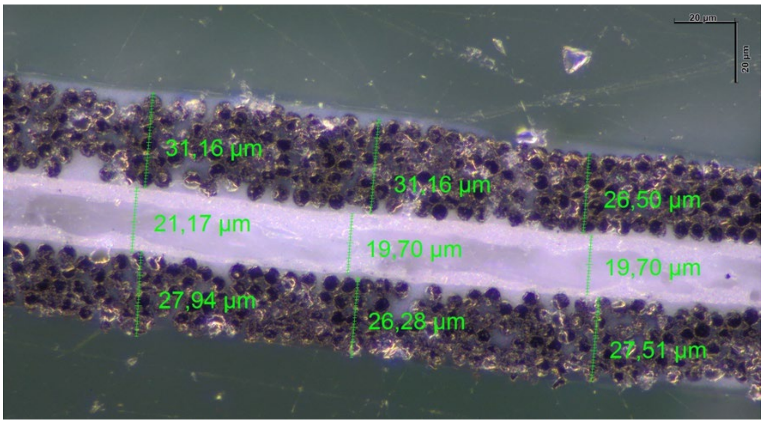


**Figure S4**. Example cross-section of laminate with measurements

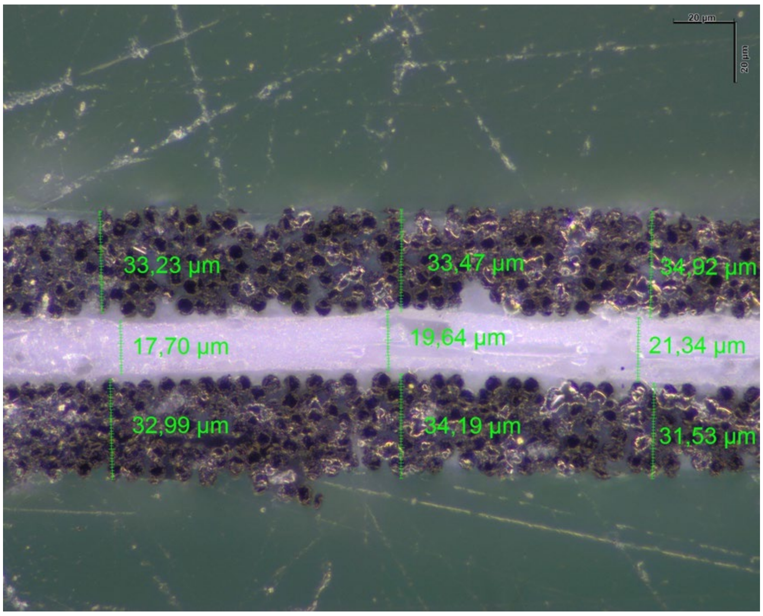


**Figure S5.** Example cross-section of laminate with measurements.

**Table S2.** Mean thicknesses from 3 different CF laminate samples, along with standard deviations.

| | **Mean CF Thickness, $t_{cf}$ (µm)** | **Standard Deviation (µm)** | **Mean Separator thickness, $t_s$ (µm)** | **Standard Deviation (µm)** |
|---|---|---|---|---|
| Sample 1 | 32,95 | 1,16 | 20,13 | 1,68 |
| Sample 2 | 29,63 | 3,28 | 21,07 | 1,38 |
| Sample 3 | 33,96 | 1,97 | 21,83 | 1,62 |

**Strain within CF laminate**

When bent, the strain varies linearly through the thickness of the composite laminate. When the laminate is bent downwards, the top CF layer is in tension (positive strain) and the bottom CF layer is in compression (negative strain), as shown in **Figure S6**. The strain in the top layer varies linearly between $\epsilon^+_{top,max}$ at the top of the layer, to $\epsilon^+_{top,min}$ at the bottom of the layer. The average strain in the layer is then $\Delta\epsilon_{33} = (\epsilon^+_{top,max} + \epsilon^+_{top,min})/2$. Similarly the average strain in the bottom layer is given as $\Delta\epsilon_{33} = (\epsilon^-_{bot,max} + \epsilon^-_{bot,min})/2$. For a constant lengthwise curvature, the average strain in the top layer is equal in magnitude but opposite in sign to the average strain in the bottom layer. The magnitude of the relative strain difference between the top and the bottom layers is therefore given as $2|\Delta\epsilon_{33}|$.

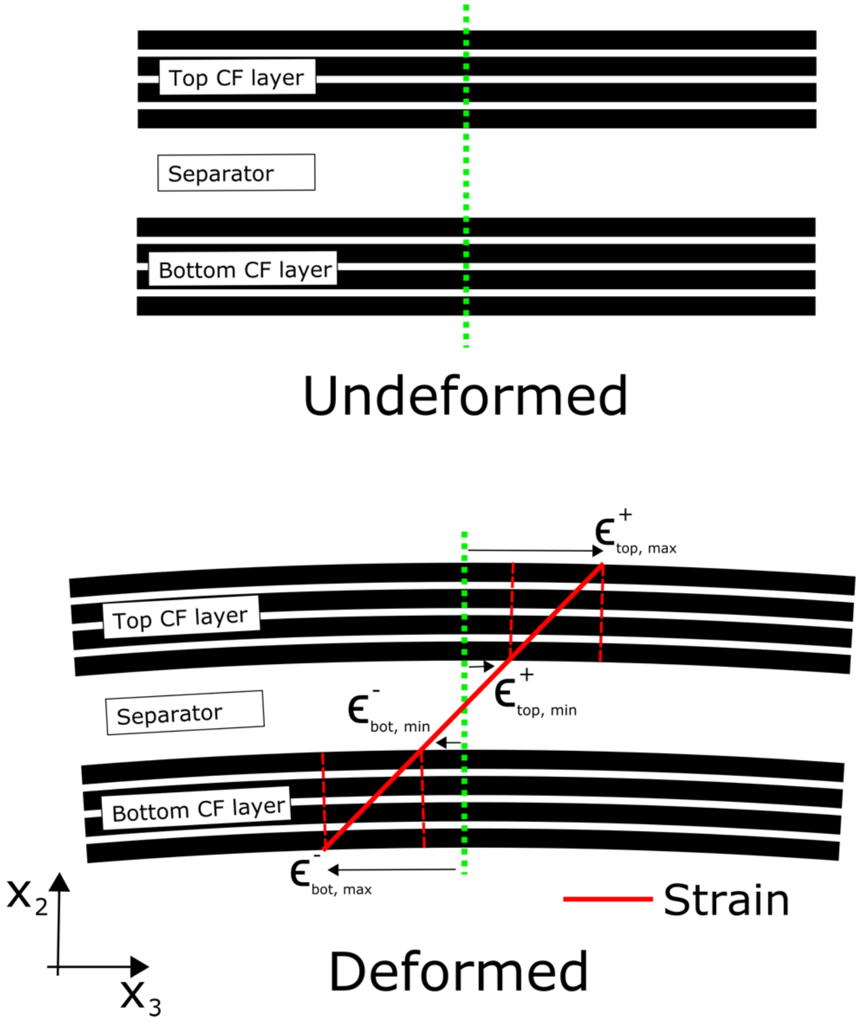


**Figure S6.** Strain variation through CF laminate thickness in undeformed and deformed states.

**Derivation of voltage-strain coupling**

The voltage-strain coupling was modelled analytically based on the assumed coupling between the chemical potential of lithium in the CFs and the mechanical stress state and is derived as follows. At equilibrium the following condition needs to be fulfilled at the electrode-electrolyte interfaces

$$\sum_i v_i \bar{\mu}_\mathrm{i} = 0 \tag{S1}$$

In eq. (S1), $v_i$ is the stoichiometric number of each species $i$ and $\bar{\mu}_\mathrm{i}$ is the electrochemical potential for each species $i$, defined as

$$\bar{\mu}_\mathrm{i} = \mu_\mathrm{i} + z_i F \varphi^\alpha \tag{S2}$$

where $F$ is Faraday's constant, $z_i$ is the valence of species $i$, $\mu_\mathrm{i}$ is the chemical potential of species $i$ and $\varphi^\alpha$ is the electric potential in phase $\alpha$. Thus, at the fiber-electrolyte interface eq. (S1) can be reformulated as

$$\bar{\mu}^\mathrm{f}_\mathrm{Li} = \bar{\mu}^\mathrm{e}_\mathrm{Li^+} + \bar{\mu}^\mathrm{f}_\mathrm{e^-} \tag{S3}$$

where $\bar{\mu}^\mathrm{f}_\mathrm{Li}$ and $\bar{\mu}^\mathrm{f}_\mathrm{e^-}$ are the electrochemical potential of Li and $\mathrm{e^-}$ in the CF, respectively. Further, $\bar{\mu}^\mathrm{e}_\mathrm{Li^+}$ is the electrochemical potential of $\mathrm{Li^+}$ in the electrolyte. Here we assume that the chemical potential of lithium in the CFs ($\mu^\mathrm{f}_\mathrm{Li}$) can be described with the Larché-Cahn potential [1,2]. This gives:

$$\begin{aligned} \mu^\mathrm{0,f}_\mathrm{Li} + RT\log\left(f^\mathrm{f}_\mathrm{Li}\tilde{c}^\mathrm{f}_\mathrm{Li}\right) - \mathrm{Tr}(\boldsymbol{\Omega\sigma}) - \tau \\ = \left(\mu^\mathrm{0,e}_\mathrm{Li^+} + RT\log\left(f^\mathrm{e}_\mathrm{Li^+}\tilde{c}^\mathrm{e}_\mathrm{Li^+}\right) + F\varphi^\mathrm{e}\right) \\ + \left(\mu^\mathrm{0,f}_\mathrm{e^-} + RT\log\left(f^\mathrm{f}_\mathrm{e^-}\tilde{c}^\mathrm{f}_\mathrm{e^-}\right) - F\varphi^\mathrm{f}\right) \end{aligned} \tag{S4}$$

where $R$ is the universal gas constant, $T$ is the temperature and $f^\mathrm{e}_\mathrm{Li^+}$, $f^\mathrm{f}_\mathrm{Li}$ and $f^\mathrm{f}_\mathrm{e^-}$ are the activity coefficients of $\mathrm{Li^+}$ in the electrolyte, Li in the fiber and $\mathrm{e^-}$ in the fiber, respectively. Further, $\mu^\mathrm{0,e}_\mathrm{Li^+}$, $\mu^\mathrm{0,f}_\mathrm{Li}$ and $\mu^\mathrm{0,f}_\mathrm{e^-}$ are the reference chemical potentials and $\tilde{c}^\mathrm{e}_\mathrm{Li^+}$, $\tilde{c}^\mathrm{f}_\mathrm{Li}$ and $\tilde{c}^\mathrm{f}_\mathrm{e^-}$ are the normalized concentrations. For the fiber we define $\tilde{c}^\mathrm{f}_\mathrm{Li} = c^\mathrm{f}_\mathrm{Li}/c^\mathrm{f}_\mathrm{Li,max}$ where $c^\mathrm{f}_\mathrm{Li}$ is the lithium concentration in the fiber and $c^\mathrm{f}_\mathrm{Li,max}$ is the assumed maximum lithium concentration in the fiber. We set $c^\mathrm{f}_\mathrm{Li,max} = \frac{1}{F}C_\mathrm{f}\rho_\mathrm{f}3600$ where $C_\mathrm{f}$ is the specific capacity of the CF and $\rho_\mathrm{f}$ is the fiber density. Further, $\boldsymbol{\sigma}$ is the mechanical stress matrix and $\boldsymbol{\Omega}$ represents the normalized

change in volume as function of the lithium concentration (commonly referred to as the partial molar volume) and is defined as

$$\boldsymbol{\Omega} = \frac{1}{c_{\mathrm{Li,max}}}\begin{bmatrix} \alpha_{\perp} & 0 & 0 & 0 & 0 & 0 \\ 0 & \alpha_{\perp} & 0 & 0 & 0 & 0 \\ 0 & 0 & \alpha_{\parallel} & 0 & 0 & 0 \\ 0 & 0 & 0 & 0 & 0 & 0 \\ 0 & 0 & 0 & 0 & 0 & 0 \\ 0 & 0 & 0 & 0 & 0 & 0 \end{bmatrix} \tag{S5}$$

where $\alpha_{\perp}$ and $\alpha_{\parallel}$ represent the normalized fiber expansion perpendicular and parallel to the fiber direction, respectively. Hence, we assume that the molar volume of the hosting compound (i.e. the CFs) is equal to $c_{\mathrm{Li,max}}^{-1}$ while $\alpha_{\perp}$ and $\alpha_{\parallel}$ correspond to the Vegard's parameters in the principal directions.[1,2] Rearranging eq. (S4) and assuming that the electron density in the fiber is effectively constant gives

$$\begin{aligned} &\mu_{\mathrm{Li}}^{0,\mathrm{f}} - \mu_{\mathrm{e}^-}^{0,\mathrm{f}} + F(\varphi^{\mathrm{f}} - \varphi^{\mathrm{e}}) + RT\log\left(f_{\mathrm{Li}}^{\mathrm{f}}\tilde{c}_{\mathrm{Li}}^{\mathrm{f}}\right) - \mathrm{Tr}(\boldsymbol{\Omega}\boldsymbol{\sigma}) - \tau \\ &\qquad = \mu_{\mathrm{Li}^+}^{0,\mathrm{e}} + RT\log\left(f_{\mathrm{Li}^+}^{\mathrm{e}}\tilde{c}_{\mathrm{Li}^+}^{\mathrm{e}}\right) \end{aligned} \tag{S6}$$

The total equilibrium potential of the CF versus lithium metal half-cell is defined as the potential difference between the electrodes given as

$$V_0 = \left[\varphi^{\mathrm{f}} - \varphi^{\mathrm{e,f}}\right] + \left[\varphi^{\mathrm{e,Li}} - \varphi^{\mathrm{Li}}\right] \tag{S7}$$

where $\varphi^{\mathrm{f}}$ and $\varphi^{\mathrm{Li}}$ are the electric potentials in the CF and lithium metal electrode, respectively. Further, $\varphi^{\mathrm{e,f}}$ and $\varphi^{\mathrm{e,Li}}$ are the electric potentials in the electrolyte at the interface towards the fiber and lithium metal, respectively. We can now define the equilibrium condition for the half-cell by combining eqs. (S6) and (S7) as

$$FV_0 = \mu_{\mathrm{Li}^+}^{\mathrm{e}} - \mu^{\mathrm{f}} + F\left[\varphi^{\mathrm{e,Li}} - \varphi^{\mathrm{Li}}\right] \tag{S8}$$

where $\mu_{\mathrm{Li}^+}^{\mathrm{e}}$ represents the chemical potential of the $Li^+$ in the electrolyte which can be expressed as

$$\mu_{\mathrm{Li}^+}^{\mathrm{e}} = \mu_{\mathrm{Li}^+}^{0,\mathrm{e}} + RT\log\left(f_{\mathrm{Li}^+}^{\mathrm{e}}\tilde{c}_{\mathrm{Li}^+}^{\mathrm{e}}\right) \tag{S9}$$

In eq. (S8), $\mu^{\mathrm{f}}$ corresponds to the part associated with the chemical potential of lithium and $e^-$ in the CFs which reads

$$\mu_{\mathrm{Li}}^{\mathrm{f}} = \mu_{\mathrm{Li}}^{0,\mathrm{f}} - \mu_{\mathrm{e}^-}^{0,\mathrm{f}} + RT\log\left(f_{\mathrm{Li}}^{\mathrm{f}}\tilde{c}_{\mathrm{Li}}^{\mathrm{f}}\right) - \mathrm{Tr}(\boldsymbol{\Omega}\boldsymbol{\sigma}) - \tau \tag{S10}$$

Here, the term $\tau$ (associated with the variation in elastic compliance with lithium concentration) is neglected since negligible change in lithium concentration is assumed.

Under the assumption of constant lithium concentration in the fiber and electrolyte, the change in equilibrium potential of the CF versus Li-metal half-cell, eq. (S8) can be defined in terms of the change in the mechanical stress state ($\Delta\boldsymbol{\sigma}$) as

$$\Delta V_0 = \frac{1}{F}\mathrm{Tr}(\boldsymbol{\Omega}\Delta\boldsymbol{\sigma}) \tag{S11}$$

Assuming that the Li concentration in the fiber is unaffected by the mechanically induced deformation, the change in stress state is reformulated in terms of the change in strain state ($\Delta\boldsymbol{\epsilon}$) as

$$\Delta\boldsymbol{\sigma} = \boldsymbol{E}\Delta\boldsymbol{\epsilon} \tag{S12}$$

where $\boldsymbol{E}$ is the elasticity matrix that is pertinent to transverse isotropy (explicit definition provided in eq. (S14)). It should be noted that small strain levels are assumed and that Voigt notations are used.

Based on eqs. (S11) and (S12) a simplified analytical expression to estimate the voltage-strain coupling of lithiated CF layers under the given uniaxial strain state is derived. The applied strain in the fiber direction ($\Delta\epsilon_{33}$) is mainly carried by the CFs and the fiber deformation in the radial direction is assumed unconstrained (motivated by the difference in stiffness between CF vs. SBE). The change in equilibrium potential of the half-cell can then be written as

$$\Delta V_0 = \frac{\Delta\epsilon_{33}}{C_\mathrm{f}\rho_\mathrm{f}3600}\left(\alpha_\parallel\left(1-\nu_{\mathrm{f},\perp}^2\right)\lambda E_{\mathrm{f},\parallel}\right) \tag{S13}$$

where $\lambda = \frac{1}{(1+\nu_{\mathrm{f},\perp})(1-\nu_{\mathrm{f},\perp}-2Y\nu_{\mathrm{f},\parallel}^2)}$ and $Y = \frac{E_{\mathrm{f},\perp}}{E_{\mathrm{f},\parallel}}$. The elastic moduli and Poisson's ratio of the fiber parallel and perpendicular to the fiber direction are denoted $E_{\mathrm{f},\parallel}$, $E_{\mathrm{f},\perp}$ and $\nu_{\mathrm{f},\parallel}$, $\nu_{\mathrm{f},\perp}$, respectively.

**Symbols and parameters**

Symbols and parameters used in the analysis presented in this paper are listed in Table S3. Moreover, the explicit representation of the elasticity matrix (for transverse isotropy) is defined as

$$
\boldsymbol{E} = \begin{bmatrix} (1-Y\nu_{\parallel}^2)\lambda E_{\perp} & (\nu_{\perp}+Y\nu_{\parallel}^2)\lambda E_{\perp} & \nu_{\parallel}(1+\nu_{\perp})\lambda E_{\perp} & 0 & 0 & 0 \\ (\nu_{\perp}+Y\nu_{\parallel}^2)\lambda E_{\perp} & (1-Y\nu_{\parallel}^2)\lambda E_{\perp} & \nu_{\parallel}(1+\nu_{\perp})\lambda E_{\perp} & 0 & 0 & 0 \\ \nu_{\parallel}(1+\nu_{\perp})\lambda E_{\perp} & \nu_{\parallel}(1+\nu_{\perp})\lambda E_{\perp} & (1-\nu_{\perp}^2)\lambda E_{\parallel} & 0 & 0 & 0 \\ 0 & 0 & 0 & 2G_{\parallel} & 0 & 0 \\ 0 & 0 & 0 & 0 & 2G_{\parallel} & 0 \\ 0 & 0 & 0 & 0 & 0 & \frac{E_{\perp}}{(1+\nu_{\perp})} \end{bmatrix} \quad \text{(S14)}
$$

where $\lambda = \frac{1}{(1+\nu_{\perp})(1-\nu_{\perp}-2Y\nu_{\parallel}^2)}$ and $Y = \frac{E_{\perp}}{E_{\parallel}}$. Further, $E_{\parallel}$, $E_{\perp}$ and $\nu_{\parallel}$, $\nu_{\perp}$ are the elastic moduli and Poisson's ratio parallel and perpendicular to the fiber direction, respectively.

**Table S3**. Material parameter values.

| Parameter | Value | Unit | Description | Reference |
|---|---|---|---|---|
| $E_{\mathrm{f},\parallel}$ | 294 | GPa | Elastic modulus of CF in the fiber direction | [3,4] |
| $E_{\mathrm{f},\perp}$ | 22 | GPa | Elastic modulus of CF perpendicular to the fiber direction | [3] |
| $\nu_{\mathrm{f},\parallel}$, $\nu_{\mathrm{f},\perp}$ | 0.2/0.2 | - | Poisson's ratio fiber | [3] |
| $C_{\mathrm{f}}$ | 163 | Ah/kg | Specific capacity of CF | [5] |
| $\rho_{\mathrm{f}}$ | 1800 | $kg/m^3$ | Fiber density | [6] |
| $\alpha_{\parallel}$ | 0.0025 | - | Li insertion induced expansion coefficient in fiber direction | [5] |
| $\alpha_{\perp}$ | 0.012 | - | Li insertion induced expansion coefficient perpendicular to fiber direction | [5] |
| $F$ | 96485 | C/mol | Faraday's constant | - |

**Direct tensile measurement of voltage-strain coupling**

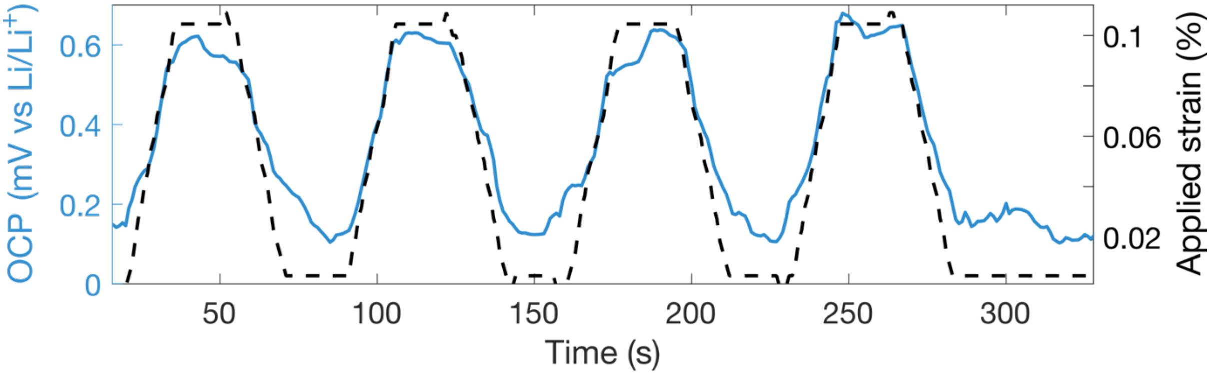


**Figure S7.** Measurement of the voltage-strain coupling using a direct tensile strain of 0.11% applied by a microtester on a CF tow, in liquid electrolyte, in a pouch cell bag against lithium metal.

The tensile specimens were manufactured using the methodology described previously.[7,8] End-tabs were attached to sections of CF using epoxy adhesive. A sheet of glass fiber separator paper was attached between the tabs. The resulting samples had a gauge length of 22 mm. These samples were dried overnight in a vacuum oven at 60°C, before being vacuum sealed in a pouch cell bag with a Li metal counter-electrode and 0.19 ml of the same electrolyte as used in the liquid phase of the SBE (1.0 M LiTf in EC:PC 1:1 wt/wt). These samples were mounted in a Deben UK microtester and connected to the potentiostat, and a mechanical strain was applied. Due to the viscoelastic pouch cell bag, the sample was then left to relax for 6 hours to allow the stress-relaxation of the bag to approach linearity. This made it straightforward to separate the force variation from the pouch cell bag and from the lithiation expansions. A current was then applied to charge and discharge the CFs. **Figure S7** shows an example OCP profile from tensile testing with an applied strain of 0.11%.

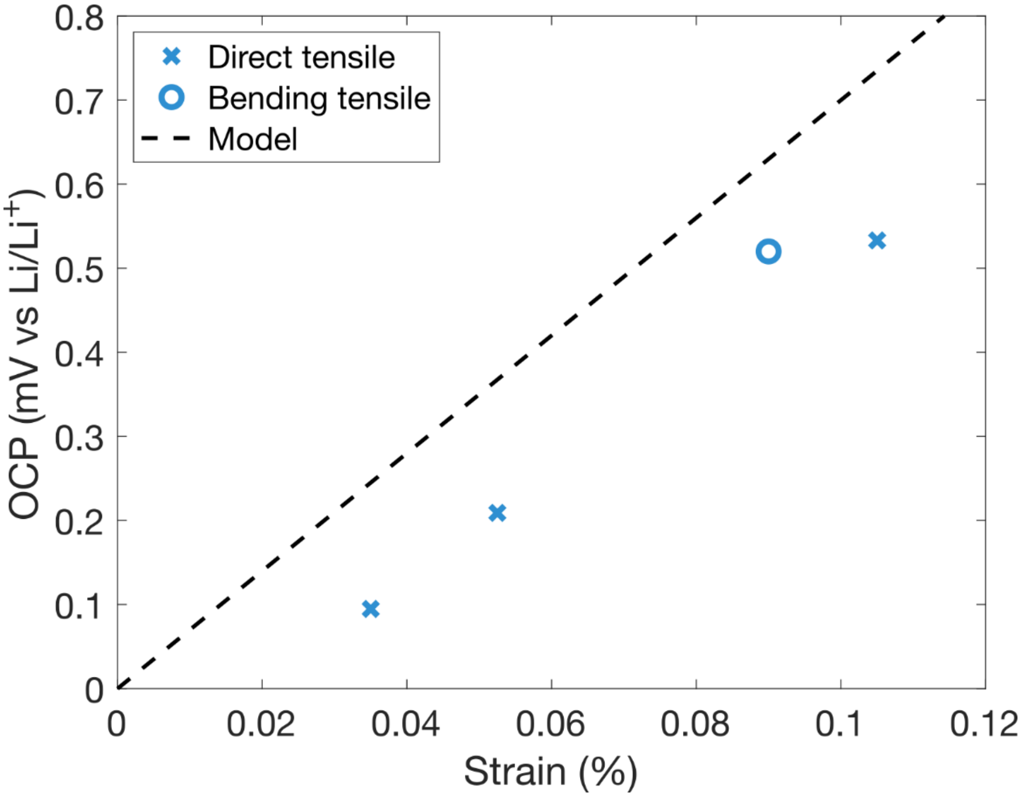


**Figure S8.** Comparison of voltage-strain coupling measurement using bend testing, and direct tensile measurements.

At the appropriate degree of lithiation, the current was stopped and the cell was left to rest for 1 hour to allow the voltage to stabilize. Cyclic mechanical tensile strains of 0.035%, 0.053%, 0.105%, were applied using the microtester at a strain-rate of 0.1 mm/min, while measuring the open-circuit potential. The voltage signals were analyzed using a fast-Fourier transform to extract the amplitude. This method was used for consistency with previous experimentation, and as a straightforward way to extract the amplitudes of voltage signal changes.[8,9]
**Figure S8** shows the experimental OCP values for tensile testing, bend testing, and predicted from the modelling.

**Electrical Impedance Spectroscopy**

Electrical impedance spectroscopy (EIS) was carried out in order to determine the internal resistance between the two CF layers. EIS measurements were carried out potentiostatically using a Gamry G750 potentiostat. The impedance was measured across the frequency range 100 kHz – 10 mHz, with an amplitude of 10 mV. **Figure S9** shows the resulting Nyquist plot. The internal impedance of the cell can be approximated using the intercept at lower frequencies. This corresponds to approximately 860 Ω.

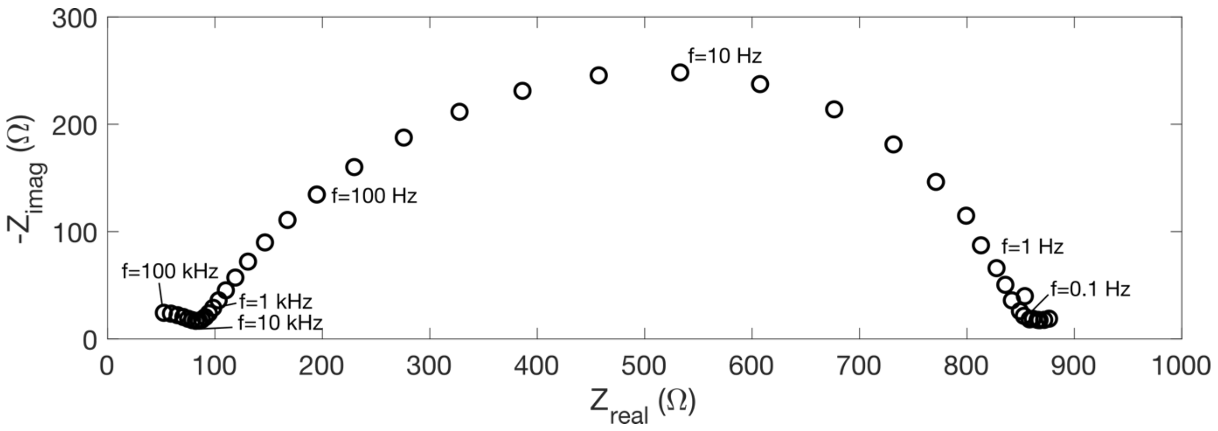


**Figure S9.** Nyquist plot following EIS measurements carried out between CF layers. The intercept at low frequency can be used to estimate the internal resistance of the cell, and in this case corresponds to approximately 860 Ω.

**Supplementary movies**

**Movie S1.** Video showing the cantilever being deformed in the downward direction to a radius of curvature of 30 mm, corresponding to an average strain magnitude of 0.09%. The OCP measured between the upper and lower CF layers versus a lithium metal reference electrode is shown. This allows the tensile and compressive OCP responses to be observed respectively. The OCP between the two CF layers is also shown, corresponding to an average strain difference of 0.18%. The first four deformations are held for approximately 25 seconds, with 25 seconds in between each deformation. The last deformation is held for approximately 120 seconds. The video is shown in real time for the first deformation, and then sped up by 10 times for subsequent deformations.

**Movie S2.** Video showing the OCP between the two CF layers for various deformations. A potentiostat is used to measure the voltage difference between the CF layers. Clamping jigs that result in an average strain difference between the CF layers of 0.04%, 0.06%, 0.08% and 0.18% are used. Three repetitions of each strain are carried out, with deformations held for approximately 10 seconds, before being released for 10 seconds. Downward bending of the cantilever is shown first, followed by upward bending. The video is shown in real time for the first three deformations, and is then sped up by 10 times for subsequent deformations.

**Movie S3.** Video showing the SCC between the two CF layers for various deformations. A potentiostat is used to hold the voltage difference between the CF layers at 0 V, and measure current. Clamping jigs that result in an average strain difference between the CF layers of 0.04%, 0.06%, 0.08% and 0.18% are used. Three repetitions of each strain are carried out, with deformations held for approximately 10 seconds, before being released for 10 seconds.

Downward bending of the cantilever is shown first, followed by upward bending. The video is shown in real time for the first three deformations, and is then sped up by 10 times for subsequent deformations.

**References**


(1) Larché, F.; Cahn, J. W. A Linear Theory of Thermochemical Equilibrium of Solids under Stress. *Acta Metallurgica* **1973**, *21* (8), 1051–1063. https://doi.org/10.1016/0001-6160(73)90021-7.

(2) Larché, F. C.; Cahn, J. W. Overview No. 41 The Interactions of Composition and Stress in Crystalline Solids. *Acta Metallurgica* **1985**, *33* (3), 331–357. https://doi.org/10.1016/0001-6160(85)90077-X.

(3) Duan, S.; Liu, F.; Pettersson, T.; Creighton, C.; Asp, L. E. Determination of Transverse and Shear Moduli of Single Carbon Fibres. *Carbon* **2020**, *158*, 772–782. https://doi.org/10.1016/j.carbon.2019.11.054.

(4) Kjell, M. H.; Zavalis, T. G.; Behm, M.; Lindbergh, G. Electrochemical Characterization of Lithium Intercalation Processes of PAN-Based Carbon Fibers in a Microelectrode System. *Journal of The Electrochemical Society* **2013**, *160* (9), A1473–A1481. https://doi.org/10.1149/2.054309jes.

(5) Jacques, E.; Hellqvist Kjell, M.; Zenkert, D.; Lindbergh, G.; Behm, M. Expansion of Carbon Fibres Induced by Lithium Intercalation for Structural Electrode Applications. *Carbon* **2013**, *59*, 246–254. https://doi.org/10.1016/j.carbon.2013.03.015.

(6) Torayca. *T800S Data Sheet*; 2015.

(7) Jacques, E.; Lindbergh, G.; Zenkert, D.; Leijonmarck, S.; Kjell, M. H. Piezo-Electrochemical Energy Harvesting with Lithium-Intercalating Carbon Fibers. *ACS Applied Materials & Interfaces* **2015**, *7* (25), 13898–13904. https://doi.org/10.1021/acsami.5b02585.

(8) Harnden, R.; Peuvot, K.; Zenkert, D.; Lindbergh, G. Multifunctional Performance of Sodiated Carbon Fibers. *Journal of The Electrochemical Society* **2018**, *165* (13), B616–B622. https://doi.org/10.1149/2.0971813jes.

(9) Harnden, R.; Zenkert, D.; Lindbergh, G. Potassium-Insertion in Polyacrylonitrile-Based Carbon Fibres for Multifunctional Energy Storage, Morphing, and Strain-Sensing. *Carbon* **2021**, *171*, 671–680. https://doi.org/10.1016/j.carbon.2020.09.042.
